# JLIN: A java based linkage disequilibrium plotter

**DOI:** 10.1186/1471-2105-7-60

**Published:** 2006-02-09

**Authors:** Kim W Carter, Pamela A McCaskie, Lyle J Palmer

**Affiliations:** 1 Laboratory for Genetic Epidemiology, Western Australian Institute for Medical Research and UWA Centre for Medical Research, University of Western Australia, Nedlands, 6009, Western Australia; 2 School of Population Health, University of Western Australia, Nedlands, 6009, Western Australia

## Abstract

**Background:**

A great deal of effort and expense are being expended internationally in attempts to detect genetic polymorphisms contributing to susceptibility to complex human disease. Techniques such as Linkage Disequilibrium mapping are being increasingly used to examine and compare markers across increasingly large datasets. Visualisation techniques are becoming essential to analyse the ever-growing volume of data and results available with any given analysis.

**Results:**

JLIN (Java LINkage disequilibrium plotter) is a software package designed for customisable, intuitive visualisation of Linkage Disequilibrium (LD) across all common computing platforms. Customisation allows the user to choose particular visualisations, statistical measures and measurement ranges. JLIN also allows the user to export images of the LD visualisation in several common document formats.

**Conclusion:**

JLIN allows the user to visually compare and contrast the results of a range of statistical measures on the input dataset(s). These measures include the commonly used D' and r^2 ^statistics and empirical p-values. JLIN has a number of unique and novel features that improve on existing LD visualisation tools.

## Background

A great deal of effort and expense are being expended internationally in attempts to detect genetic polymorphisms contributing to susceptibility to complex human disease. Concomitantly, the technology for detecting and scoring single nucleotide polymorphisms (SNPs) has undergone rapid development, yielding extensive catalogues of SNPs across the genome. Population-based maps of the correlations amongst SNPs (linkage disequilibrium) are now being developed with the aim to accelerate the progress of complex human gene discovery. A growing problem in complex disease genetics is the sheer volume of SNP data being generated in gene discovery projects. With such large volumes of data available, it is essential to have the ability to examine results in a graphical form rather than text [1].

Linkage Disequilibrium (LD) is a statistical measure of the non-independence of alleles at adjacent loci. Two markers having alleles that are correlated with each other in a population are said to be in LD. Such loci are generally in close physical proximity, but the relationship can vary dramatically. When a new variant is first introduced into a population (by mutation) it will be perfectly correlated with nearby variants. Over successive generations the process of meiotic recombination will break down the correlations among nearby variants, and thus LD decays. Markers that are in 'perfect' LD with each other (i.e., having a statistical correlation of 1.0) are entirely redundant in the sense that an individual's genotype at one locus will completely predict that at the other locus. Conversely, markers that show no LD are statistically independent and convey no information about each other, even if they are in extremely close physical proximity. The indirect association mapping model that is the current paradigm for gene discovery in complex human disease relies on LD in the sense that the functional variant need not be studied at all, so long as one measures a variant that is in LD with it. We have developed a visualisation tool, referred to as Java LINkage disequilibrium plotter (JLIN), to aid researchers in performing LD analysis.

## Implementation

JLIN is written in Java to enable cross-platform support, and is downloadable with a Java installer. JLIN has been tested on datasets ranging in size from several markers to in excess of 100 markers. JLIN is only limited by machine speed and memory size and has been tested on several hundred markers. While JLIN has been tested on datasets containing nearly one thousand markers, we note that it is highly unlikely that a researcher will be looking for pairwise LD across thousands of markers as this implies a larger region than LD would normally extend across in an outbred population.

Coping with missing genotype data is an important and common problem when dealing with genetic datasets. JLIN handles missing data by examining which SNP genotypes for each individual contain missing data. Rather than ignoring individuals with missing data, JLIN only ignores a particular individual's data for pairwise LD comparisons where one or both of the SNPs contain missing data. This way, for all pairwise SNP comparisons with no missing data, the data for each particular individual is fully utilised.

## Results

JLIN is a customisable, intuitive LD visualisation tool. As no single LD measure appears to be the best for all circumstances [2-4], JLIN allows the user to visually compare and contrast the results of a range of LD statistical measures. The LD statistics calculated are D, D', r^2^, OR, P_excess_, d and Q, as described by Devlin and Risch [2], along with Hardy Weinberg Equilibrium calculations for each SNP marker [5]. In addition, JLIN has the ability to calculate empirical p-values for the pairwise association of two SNPs, as described by Slatkin and Excoffier [6], another unique feature amongst LD visualisation tools.

We have developed a simple, intuitive interface that enables the user to customise the results presented. JLIN allows the user to visualise one or two LD statistics in a single display (user controlled) along with the ability to export the display into three common publishing formats, namely portable document format (pdf), encapsulated postscript (eps) and portable network graphics (png). JLIN accepts genotype data in a simple comma-separated value (CSV) input file and imputes haplotypes (currently for bi-allelic markers) using an expectation-maximisation algorithm (EM) [7]. A visual representation of physical distance between markers is also available (distances are supplied in the input CSV file). In addition JLIN has the ability to calculate empirical p-values (derived from conducting multiple permutations of data), a unique feature among freely available and commercial LD analysis tools. The user has the flexibility to select different colour schemes (including black and white), along with the ability to change the minimum, maximum and increment values independently for each of the statistics shown. Future extensions to JLIN will include calculating multi-locus haplotypes, imputation of missing genotype data and handling multi-allelic markers.

A number of freely available and commercially released LD visualisation tools are available. GOLD [8] has a rather distinct display format that is perhaps its strength and major weakness, in addition to being primarily Windows based (for the graphical interface). LDA [9] and Haploview [10] are written in Java, to enable cross-platform support, and implement a number of LD measures, but LDA allows little flexibility or user control over the interface and presentation of results. GOLD and Haploview do provide several features which are beyond the scope of JLIN currently, such as the ability to utilise family data for haplotypes estimation and the estimation of haplotype tagging SNPs. Helixtree [11] is similarly designed in Java, and while it has numerous features, is both commercial software and only freely available as a trial version. JLIN introduces a number of unique features in terms of statistical calculation and presentation, and adds flexibility and customisation for the user that does not appear in existing LD visualisation tools.

## Conclusion

JLIN is a novel and intuitive visualisation tools designed to give the user capability and flexibility for LD analysis. JLIN implements a wide range of statistical measures and analysis methods, coupled with export options and a range of features that forms a unique integrated analysis package.

## Availability and requirements

**Project name: **JLIN: A java based linkage disequilibrium plotter 

**Project home page: **

**Operating system(s): **Platform independent

**Programming language: **Java

**Other requirements: **Java 1.5.0 or higher

**License: **Free for non-commercial use

**Any restrictions to use by non-academics: **Please contact authors

**Figure 1:**
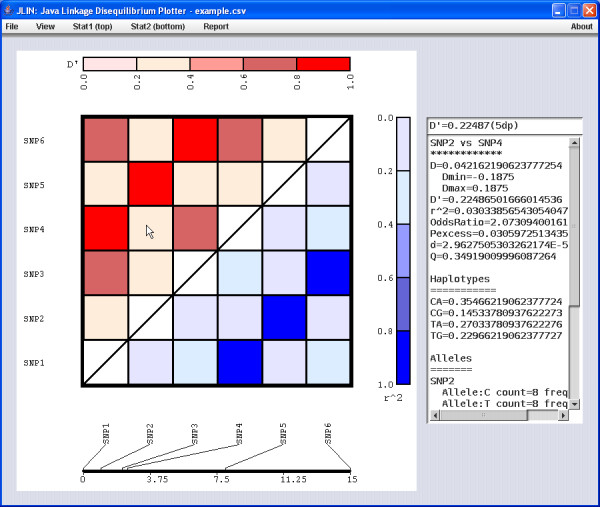
**JLIN screenshot**. Figure 1 shows the JLIN visualisation for the pairwise LD comparison of six SNP markers, labelled SNP1 to SNP6, within a single gene. The top left triangle of the display (red triangle area) shows the pairwise D' LD statistics, while the bottom right triangle (blue) shows the pairwise r^2 ^statistics. Below this is a display measure to indicate relative physical distance between the markers. By selecting a particular comparison square, all available statistics for the particular comparison are displayed in the information area on the right of the graphical display. In Figure 1, the D' comparison between SNP2 and SNP4 was selected, with full statistics of the comparison between the two SNPs, including each possible haplotype and their associated calculated frequency, allele counts and frequencies for each SNP and genotype counts and frequencies for each SNP.

## Authors' contributions

KWC designed and developed the Java implementation of the underlying algorithms and GUI. PAM designed the statistical analysis framework and aided with design of the GUI. LJP conceived of the software and participated in the design and coordination of its development.
